# ROS induced lipid peroxidation and their role in ferroptosis

**DOI:** 10.3389/fcell.2023.1226044

**Published:** 2023-08-01

**Authors:** Hiwot Tezera Endale, Winta Tesfaye, Tiget Ayelgn Mengstie

**Affiliations:** ^1^ Department of Biochemistry, School of Medicine, College of Medicine and Health Sciences, University of Gondar, Gondar, Ethiopia; ^2^ Department of Human Physiology, School of Medicine, College of Medicine and Health Sciences, University of Gondar, Gondar, Ethiopia

**Keywords:** ROS, lipid peroxidation, autophagy, ferroptosis, apoptosis

## Abstract

Reactive oxygen species (ROS) play a crucial part in the process of cell death, including apoptosis, autophagy, and ferroptosis. ROS involves in the oxidation of lipids and generate 4-hydroxynonenal and other compounds associated with it. Ferroptosis may be facilitated by lipid peroxidation of phospholipid bilayers. In order to offer novel ideas and directions for the investigation of disorders connected to these processes, we evaluate the function of ROS in lipid peroxidation which ultimately leads to ferroptosis as well as proposed crosstalk mechanisms between ferroptosis and other types programmed cell death.

## Introduction

Reactive oxygen species are the byproduct of molecular oxygen which includes reactive nitrogen, sulfur, carbon, selenium, electrophile and halogen species. During normal metabolism, reactive oxygen species (ROS) are formed through both enzymatic and non-enzymatic routes. Besides, excess amounts of ROS interact with numerous biological components, such as DNA, lipids, and proteins, they are also capable of creating other reactive species that may have harmful effects (Halliwell and Gutteridge, 2015; Li et al., 2022). Due to their high levels of polyunsaturated fatty acids (PUFAs), cellular membranes or organelle membranes are particularly vulnerable to ROS damage, often known as “lipid peroxidation.” Lipid peroxidation results from oxygen-free radicals attacking polyunsaturated fatty acids, which are extremely susceptible to this, damage (Yang et al., 2016; Que et al., 2018; Liang et al., 2022). Phospholipids are directly destroyed by lipid peroxidation, which is also utilized as a cell death signal to cause intended cell death. Recent research has revealed that lipid peroxidation caused by ROS encouraged the processes of apoptosis and autophagy. Lipid peroxidation as a consequence of ROS also promotes non-apoptotic cell death, Ferroptosis, which is dependent on iron and lipid reactive oxygen species, amplifies lipid peroxidation’s role in the biological process of cell death (Yang et al., 2016; Chen et al., 2017; Zhong et al., 2017; Yu et al., 2021). Understanding how ROS are generated and the mechanism lipid peroxidation driven by ROS contributes to cell death is crucial. In this piece, we provide an overview of the mechanism of ROS-induced lipid peroxidation in ferroptosis and discuss about how ferroptosis interact with programmed cell death (apoptosis and autophagy).

### Reactive oxygen species: friend or foe?

Free radicals are examples of partially reduced oxygen-containing molecules known as ROS. Superoxide radical, which is produced primarily by NADPH oxidases (NOXs), xanthine oxidase (XO), and the mitochondrial electron-transport chain (mETC) in endogenous biologic systems, is the source of the majority of intracellular ROS. Through the Fenton reaction, which uses various peroxide species to produce hydroxyl (OH) or alkoxyl (RO) radicals, ROS are transformed by the superoxide dismutase (SOD) to hydrogen peroxide, which then releases the highly deadly hydroxyl radical in the presence of reduced iron (Fe2+). Through the Haber-Weiss reaction, ferric iron (Fe3+) can be converted back to Fe2+ by oxidizing with a peroxyl radical and oxygen. Oxidative stress is caused by an imbalance in the rate of ROS synthesis, which results in a generation of free radicals that may attack DNA, proteins, and lipids (Kruszewski, 2003; Sakellariou et al., 2014; Latunde-Dada, 2017). ROS may operate as signaling molecules, which played crucial roles in modulating a variety of physiological processes, including the growth, proliferation, differentiation, autophagy, and death of cells. ROS formation and removal were subject to strict supervision. In the meantime, cells went through various stages of life on a regular basis. The proliferation of cells was markedly accelerated by H_2_O_2_ (1–10 M) treatment. When Jurkat T-lymphocytes were exposed to 50 M H_2_O_2_, the induction of apoptosis was sluggish. Although by 6 h, recognizable apoptotic alterations were apparent. Contrarily, cells could undergo direct necrosis in response to a high level of oxidative stress (100 M H_2_O_2_). Surprisingly, studies have shown that the H_2_O_2_ that human colon cancer cells create may play a crucial role in triggering cell death to produce anti-tumor effects (Zeng et al., 2023). The dynamic and stable balance of ROS depends heavily on the antioxidant system of the cell. The ability of this antioxidant system to maintain equilibrium *in vivo* depends on maintaining levels of hydrogen peroxide and nitric oxide (NO) production and elimination within a range that prevents the buildup of significant amounts of peroxynitrite (ONOO) or hydroxyl radical (Forman et al., 2014; Ferreira et al., 2018). endogenous antioxidants consist of both non-enzymatic, such as vitamins or their analogues (vitamins A, C, and E; coenzyme Q10; flavonoids), minerals (selenium and zinc), and metabolites (bilirubin and melatonin). Enzymatic antioxidants include superoxide dismutase, catalases, glutathione peroxidase (GPX), glutathione reductase. Oxidative stress can be brought on by redox state changes and antioxidant depletion, which can result in oxidative damage (Sies et al., 2017; Liguori et al., 2018).

### Reactive oxygen species: induction of cell damage

Numerous biological components, including DNA, lipids, and proteins, interact with ROS (Halliwell and Gutteridge, 2015). Base alterations, abasic sites, and single- or double-stranded breaks in DNA are only a few of the several DNA lesions that can result from oxidative stress. According to Srinivas et al., 2019, the main way that DNA gets modified is through the 8-hydroxylation of guanine (8-hydroxy-2′-deoxyguanosine). Protein carbonyl formation and mercaptan oxidation are the two most frequent oxidative changes of proteins. Protein oxidation reduces an enzyme’s ability to bind and function. The sulfur-containing amino acids cysteine and methionine are more likely to be attacked by oxidative processes. Methionine and cysteine can both be oxidized to produce methionine sulfoxide and sulfur radical and disulfides respectively (Ahmad et al., 2017; Colovic et al., 2018). Polyunsaturated fatty acids, which are significant phospholipids in cell membranes, control the fluidity and deformability of the cell membrane (Mondal et al., 2019). Lipid peroxidation results from assaulting polyunsaturated fatty acids by oxygen free radical, which are extremely susceptible. The creation of lipid-free radicals results in the formation of peroxidized-free radicals, which target nearby polyunsaturated fatty acids and membrane proteins while also causing membrane lipid peroxidation (Yang et al., 2016; Que et al., 2018; Liang et al., 2022).

### Role of reactive oxygen species in lipid peroxidation

In broad terms, the process of oxidants like free radicals attacking lipids with carbon-carbon double bonds, notably polyunsaturated fatty acids (PUFAs), is known as lipid peroxidation (Yang et al., 2016). Three steps make up the lipid peroxidation process mediated by ROS: initiation, transmission, and termination. In the first phase of lipid peroxidation, promoters like hydroxyl radicals remove allyl hydrogen to produce lipid-free radicals with a carbon nucleus. During the transmission stage, lipid-free radicals quickly combine with oxygen to form lipid peroxy radicals, which then continuously chain-react to make new lipid free radicals and lipid hydrogen peroxide by stealing hydrogen from more lipid molecules. Antioxidants, including vitamin E, give lipid peroxy radicals a hydrogen atom in the termination reaction. This results in the formation of a vitamin E-free radical, which then combines with another lipid peroxy radical to create a non-free radical product. Lipid peroxidation starts a chain reaction that continues until the termination product is created (Hermetter et al., 2012; Volinsky and Kinnunen, 2013; Que et al., 2018). Lipid hydro-peroxides are the major end result of lipid peroxidation. Furthermore, it has been found that a variety of aldehydes, including malondialdehyde (MDA), propionaldehyde, hexanal, and 4-hydroxynonenal (4HNE), were produced as byproducts of lipid peroxidation. Of them, 4HNE has been demonstrated to play a significant role as a signaling molecule, promoting gene expression, improving cellular antioxidant capacity, and enhancing adaptive responses at low levels. It can also induce autophagy, senescence, or cell cycle arrest upon organelle and protein damage at moderate levels. And at high or extremely high levels, it can promote adduct formation and apoptosis or necrotic cell death (Zarkovic, 2003; Ayala et al., 2014; Schaur et al., 2015).

### Reactive oxygen species and ferroptosis

The word “ferroptosis” refers to a novel non-apoptotic cell death process involving iron and lipid reactive oxygen clusters. Three key characteristics of ferroptosis were observed: (Li et al., 2022): oxidation of membrane phospholipids containing PUFA; (Halliwell and Gutteridge, 2015); availability of redox active iron; and (Que et al., 2018) lack of the ability to repair lipid hydroperoxide (Dixon et al., 2012). As a result, it was shown that oxidative stress and cellular antioxidant levels were key regulators of lipid peroxidation in ferroptosis. It was brought on by the drugs that can avoid iron-dependent lipid peroxidation and by lipophilic antioxidants like vitamin E, ferrostatin-1, liproxstatin-1, and polyphenols with strong biological activity. It was also brought on by the drugs erastin, salazosulfapyridine, and RSL3 (Angeli et al., 2017; Shah et al., 2017). Numerous cellular metabolic pathways, such as cellular respiration (such as the mitochondrial tricarboxylic acid (TCA) cycle and electron transport chain), lipid metabolism, and amino acid metabolism, generate significant amounts of ROS and consequently cause ferroptosis (Stockwell et al., 2017; Javadov, 2022).

#### Mechanisms of ferroptosis

Ferroptosis involves two main players: iron and lipid peroxides. It appears that ferroptosis is ultimately caused by the buildup of lipid peroxides, particularly phosphatidylethanolamine-OOH (PE-OOH), with iron acting as a catalyst or a crucial regulator of ferroptosis. In the presence of glutathione (GSH), a cofactor of GPX4, hazardous lipid peroxides are converted to harmless lipid alcohols by GPX4. Through the removal of internal lipid ROS, GPX4 protects cells from ferroptosis, while GPX4 inhibition results in the onset of ferroptosis. Ferroptosis is also started by ROS generated by the Fenton reaction, which is accelerated by iron (Toyokuni et al., 2017; Lei et al., 2019).

#### GPX4 activity and ferroptosis

The system Xc—GSH—GPX4 route is the classic ferroptosis control axis. Glutamate cysteine ligase (GCL) and glutamate cysteine synthase (GSS), which are both catalyzed by GSH, produce cystine after the system Xc-swaps internal glutamate for extracellular cystine at a 1:1 ratio. High levels of oxidative stress make most cancer cells more vulnerable to GSH shortage, which is a weakness that can be exploited in cancer therapy. Any phospholipid hydrogen peroxide in cells is reduced by GPX4 to the corresponding alcohols by the action of GSH, a powerful reducer and cofactor for GPX4. Since GPX4 is the most significant reductase, it has been discovered that its inhibition and silencing cause ferroptosis, but its overexpression lowers ROS levels and inhibits cells from going through ferroptosis (Dixon et al., 2012; Lv et al., 2019; Bai et al., 2021; Jiang et al., 2021; Liu et al., 2021). RSL3 is a specific inhibitor of GPX4, leading to cellular ferroptosis by inhibiting GPX4 activity. FIN56 caused ferroptosis by depleting GPX4 and coenzyme Q (Dixon et al., 2012). The trace element selenium was also found to be important for GPX4 activity (Dixon et al., 2012; Hadian and Stockwell, 2020). The catalytic and regulatory subunits of GCL (GCLC and GCLM), GSS, and a subunit of the system Xc-solute carrier family 7 member 11 (SLC7A11) are essential for GSH synthesis and under the control of nuclear factor erythroid 2-related factor 2 (NRF2). NRF2 is a comprehensive regulator of the anti ferroptotic reaction since it controls the expression of GPX4 as well as other critical antioxidant defense system components (Saha et al., 2020; Akiyama et al., 2022; Li and Huang, 2022).

#### Mechanism of iron metabolism that promote ferroptosis

Lipoxygenases (LOXs) and P450, the main ROS-producing enzymes during ferroptosis, require iron as a cofactor for their biosynthesis. Through the Fenton reaction and the Haber-Weiss reaction, the iron metabolism process encourages lipid peroxidation. Exogenous iron supplementation makes cells more susceptible to drugs that inhibit ferroptosis, including erastin. The so-called “labile iron pool” (LIP), a modest amount of uncoordinated redox activity of Fe2+, is also present in cells. By lowering the availability of iron in the LIP, iron chelators and iron metabolism inhibitors (such as deferoxamine and ciclopirox) reduced lipid peroxidation (Doll and Conrad, 2017; Silva et al., 2018; Pan et al., 2022). Numerous essential iron storage, metabolism, and transport proteins, including ferritin light chain (FTL), ferritin heavy chain 1 (FTH1), SLC40A1, and biliverdin reductases (BLVRA/B), were found to be transcriptionally regulated by NRF2 as well. The breakdown of heme to ferrous iron, biliverdin, and carbon monoxide was facilitated by excessive activation of HO-1 that was controlled by NRF2, raising the iron level in the lip and promoting ferroptosis. Therefore, HO-1′s protective effect was attributed to its antioxidant activity, whereas its toxic effect of excessive upregulation was attributed to increased ferrous iron production that encouraged Fenton reaction-mediated peroxide catabolism in the presence of a deficiency in ferritin buffering capacity. Furthermore, through the accommodation of ferritin and transferrin receptors, ROS-mediated autophagy raised intracellular iron levels and ferroptosis (Kerins and Ooi, 2018; Chen et al., 2020; Li et al., 2021).

## The interaction of ferroptosis with other type of cell death in oxidative environment

### Ferroptosis and apoptosis

**FIGURE 1:**
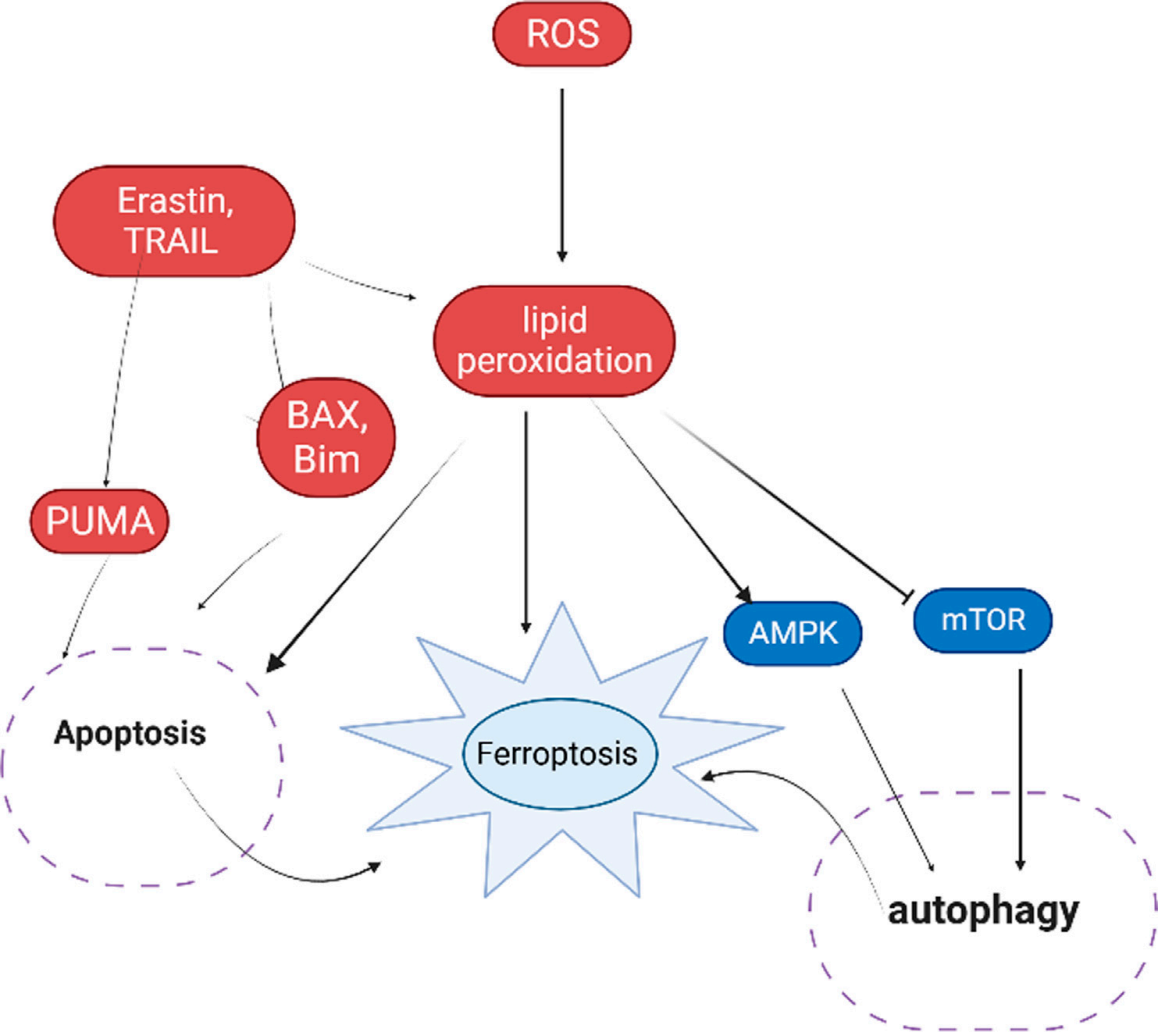
Interaction of ferroptosis with apoptosis and autophagy under oxidative environment.

There was evidence that apoptosis and ferroptosis interacted and ultimately caused cell death. NADPH oxidase (NOX) was demonstrated to be involved in the activation of apoptosis as a key regulator of lipid redox signaling (Mukherjee and Lynn, 1977). Similar to how NOX1-, NOX2-, and NOX4-mediated ROS generation caused lipid peroxidation to begin ferroptosis (Dixon et al., 2012; Xie et al., 2016; Chen et al., 2019). It was shown that ferroptosis inducers, such as erastin and artesunate, promoted P53 upregulated modulator of apoptosis (PUMA) and possibly enhanced TRAIL-induced apoptosis by increasing death receptor 5 levels as mentioned in Figure 1. These effects of the ferroptosis inducers and TRAIL to promote apoptosis were synergistic (Jiang et al., 2015; Xie et al., 2017; Lee et al., 2018; Lee et al., 2019). Cystatin protease cleaves acyl-CoA synthase long chain family member 4 (ACSL4) during bortezomib-induced apoptosis (Guo, 2022). Therefore, it is possible that the inactivation of ACSL4 during apoptosis prevents the insertion of PUFAs into the cell membrane, thereby reducing the capacity of cells to undergo ferroptosis. Apoptosis can be induced by some ferroptosis inducers via mitochondria-related pathways. Erastin and TRAIL therapy improved apoptosis via a BAX-mitochondria dependent mechanism (Lee YS. et al., 2020; Tang et al., 2022). Erastin caused cancer cells to undergo caspase-9-dependent mitochondrial apoptosis (Chen et al., 2021a). Ferroptosis caused by erastin was inhibited by the BH3 interacting domain (BID) inhibitor BI-6c9 (Battaglia et al., 2020). Bim and Bax functioned as cross-linked regulators of abivertinib-induced apoptosis and ferroptosis, activating the apoptotic pathway by upregulating caspases and the ferroptosis pathway by suppressing SLC7A11 and GPX4, both of which need mitochondria to function (Tang et al., 2022).

Bid is a GPX4 downstream signal molecule that has a role in ferroptosis in neuronal cells brought on by RSL3. While it undergoes different modifications during apoptosis and ferroptosis, the mitochondria, an essential organelle in both kinds of cell death, may be an excellent subject for research into the relationship between the two. Erastin altered the voltage-dependent anion channels (VDACs) on the mitochondrial membrane during ferroptosis. This reversed the Warburg effect and enhanced ROS formation by causing an increase in oxidative phosphorylation and a decrease in the production of ATP via glycolysis (Wilde et al., 2017; Oh et al., 2022; Zhang et al., 2022). Contrarily, during apoptosis, other BCL2 family members control the mitochondrial membrane further, discharging chemicals such cytochrome c (Dadsena et al., 2021). On the other hand, since the generation of ROS is a crucial part of mitochondria’s role in ferroptosis, while the release of chemicals like cytochrome c through ruptured membranes is a crucial part of apoptosis. Ferroptosis and apoptosis share the same signaling molecules, thus possibly this could be investigated potential interactions. Ferroptosis was discovered to be bidirectionally regulated by p53 by transcriptional or 1 3 post-translational pathways, in addition to its effects on apoptosis, autophagy, and the cell cycle. One way that p53 induced ferroptosis was by directly inhibiting the activity of dipeptidyl peptidyl peptidase 4 (DPP4) or by inducing the expression of cell cycle protein-dependent kinase inhibitor 1A (CDKN1A/p21). On the other hand, p53 promoted ferroptosis by inhibiting the expression of SLC7A11 or by increasing the expression of spermine/spermine 1 acetyltransferase 1 (SAT1) and glutaminase 2 (GLS2). Under basal or low ROS stress, P53 may function as a rheostat, inhibiting ferroptosis while boosting it under high oxidative stress. (Kang et al., 2019; Ji et al., 2022).

### Ferroptosis and autophagy

Autophagy has a role in ferroptosis. Strong inducers of autophagy include oxidative stress and the byproducts of lipid peroxidation, including MDA, ROS, and 4-HNE, while excessive autophagy encourages ferroptosis (Liu et al., 2020; Zhao et al., 2020). By destroying ferroptosis repressors in a way that boosted intracellular free iron or made it possible for the buildup of lipid peroxides, selective kinds of autophagy and chaperone-mediated GPX4 autophagy (Yu et al., 2022) promoted ferroptosis. The nuclear receptor coactivator 4 (NCOA4)-mediated degradation of intracellular ferritin was found to increase ferritinophagy by releasing free iron and encouraging ferroptosis, which was aided by ROS (Hou et al., 2016). RSL3 increased ferroptosis by raising intracellular-free lipids through the destruction of lipid droplets and aryl hydrocarbon receptor nuclear translocation factor-like (ARNTL/BMAL1), which in turn caused lipophagy and clockophagy (Liu et al., 2019; Liu et al., 2020). ROS and Erastin can encourage mitophagy, the selective autophagy of mitochondria (Basit et al., 2017). Additionally, by blocking mTOR or enhancing the AMPK pathway, certain inhibitors and medications can cause ferroptosis and autophagy. Rapamycin was discovered to act as a typical mTOR inhibitor, causing autophagy that was beneficial for survival at low dosages but autophagy-dependent ferroptosis at large levels (Wu et al., 2022). Additionally, mTOR was inhibited by cysteine deficiency (Zhou et al., 2021). Erastin increased autophagy by boosting the AMPK pathway, while RSL3 promoted autophagy by blocking mTOR activation (Figure 1) (Li et al., 2020; Gong et al., 2022). Erastin and sulfasalazine enhanced AMPK phosphorylation, which in turn promoted BECN1 phosphorylation and binding to SLC7A11, leading to GSH depletion and ferroptosis. Acetyl-CoA carboxylation (ACACA) was phosphorylated by AMPK, which inhibited it in order to reduce ferroptosis (Lee H. et al., 2020; Chen et al., 2021b; Chen et al., 2022). P62 served as an autophagy cargo receptor and activated NRF2 by deactivating the protein Kelch-like ECH-associated protein 1 (KEAP1) to prevent ferroptosis. This suggests that p62/KEAP1/NRF2 functions as a similar route to control both autophagy and ferroptosis (Lleonart et al., 2018). It is yet unknown how the autophagic machinery reacts to ferroptosis activators to change from a pro-survival to a fatal fashion.

## Conclusion

A precise balance between the production and removal of ROS is maintained in connection with a number of pathogenic processes, including cell growth, differentiation, and death. Cells are safeguarded against oxidative damage by the general endogenous antioxidant system, which consists of both enzymatic and nonenzymatic antioxidants. Cell death is caused by lipid peroxidation, which is induced by an excess of ROS. Newer types of programmed cell death include ferroptosis. It is not entirely understood how cumulative lipid peroxidation caused ferroptosis to develop. Currently, ferroptosis has been discovered to be intimately linked to a number of diseases, making it a novel method of death. If the riddle of lipid peroxidation-induced ferroptosis is solved, it may offer fresh perspectives and treatment options for various diseases in humans that are linked to ferroptosis. But, the effectiveness of a single ferroptosis inhibitor in treating various disorders has to be further investigated. Cell death was finally brought on by ferroptosis, which combined with apoptosis and autophagy. In addition, there are many studies on the interactions between ferroptosis and apoptosis and autophagy, but more research is still needed to understand how these cell death mechanisms relate to oxidative stress in order to identify possible targets for future therapies.
